# Careful conversations: an educational video to support parents in communicating about weight with their children

**DOI:** 10.1186/s12887-020-02284-6

**Published:** 2020-08-24

**Authors:** Kody A. Klupt, Stephan M. Oreskovich, Julie Bernard-Genest, Barkha P. Patel, Lisa Chu, Elizabeth Dettmer, Catharine M. Walsh, Michele Strom, Amy C. McPherson, Jonah Strub, Alissa Steinberg, Cathleen Steinegger, Jill K. Hamilton

**Affiliations:** 1 Division of Endocrinology, The Hospital for Sick Children, 555 University Ave, Toronto, Ontario M5G1X8 Canada; 2 Centre Mère-Enfant Soleil du Centre hospitalier universitaire de Québec, Université Laval, 2705 Boulevard Laurier, Québec City, Québec G1V4G2 Canada; 3 Bloorview Research Institute, Holland Bloorview Kids Rehabilitation Hospital, 150 Kilgour Rd, East York, Toronto, Ontario M4G1R8 Canada; 4 Dalla Lana School of Public Health & Rehabilitation Sciences Institute, University of Toronto, 155 College St, Toronto, Ontario M5T3M7 Canada

**Keywords:** Educational video, Communicating weight, Parents, Pediatric obesity, Healthy conversations

## Abstract

**Background:**

Parents may struggle to initiate healthy weight-related conversations with their children. Educational videos may be an effective tool for improving parents’ knowledge and self-efficacy on this topic. The aim of this pilot study was to develop an educational video to assist parents in weight-related conversations with their child, and to assess changes in parents’ self-efficacy on this topic.

**Methods:**

Video development was based on a scoping review and semi-structured interviews with parents. Respondent demographics and user satisfaction were assessed at pre- and post- video, and 4–6 months later. Self-efficacy scores were compared between parent groups based on weight concerns over time.

**Results:**

Fifty-seven parents participated in the video questionnaires, and 40 repeated measures 4–6 months later. Significant improvements in self-efficacy in “raising the issue of weight” and “answering questions or concerns” were found after watching the video (*p* ≤ 0.002) compared to baseline, and scores 4–6 months post baseline remained slightly elevated, but non-significant. Parents with concerns about their child being overweight had significantly lower perceived self-efficacy scores compared to parents with no concerns about their child’s weight (*p* = 0.031). The video was found to be positively received and of relevance to parents across a number of different domains.

**Conclusion(s):**

Preliminary findings suggest an educational video about initiating weight-related conversations may be an effective tool for increasing parents’ perceived self-efficacy in the short term. Further work is needed to validate findings in a randomized controlled trial, and with diverse parent populations.

**Trial registration:**

ClinicalTrials.gov Identifier: NCT03664492. Registered 10 September 2018 – Retrospectively registered

## Background

The prevalence of obesity amongst children in North America remains high, and has risen to over 17% [1, 2]. Further, in a population-based survey of over 10,000 adolescents between 13 and 18 years of age, lifetime prevalence of anorexia nervosa, bulimia nervosa, and binge eating, were notably prevalent with rates of 0.3, 0.9, and 1.6%, respectively [3]. While parents generally believe discussing the weight of their children is important, some parents can experience difficulties doing so, fearing that it may lower their child’s self-esteem, as stigmatization of obesity in youth may lead to harm and the development of unhealthy behaviours, such as binge eating and avoidance of health care services [4, 5]. This is reinforced by evidence suggesting that focusing on conversations involving weight or size may increase the risk of disordered eating behaviours, whereas an emphasis on growth and healthy behaviours is a more effective strategy for parents wishing to engage with their children [6, 7]. Few resources exist to guide these sensitive discussions.

Educational videos can be an effective tool to improve knowledge and self-efficacy on a given topic [8, 9]. However, the development and implementation of a video guiding parents who wish to engage in weight-related conversations with their child has not yet been studied. Accordingly, the objective of the present study is to create an educational video, and to assess the effect on parents’ self-efficacy for communicating about weight-related issues with their children. We hypothesized that the video would have a positive effect on parent’s self-efficacy with regards to raising the issue of weight with their children, and addressing questions their child may have about this topic.

## Methods

This non-randomized, prospective, single arm pilot trial was conducted at the Hospital for Sick Children (SickKids), an urban pediatric academic hospital in Toronto, Canada. Approval for the study was granted by the SickKids Institutional Research Ethics Board.

### Video development

A scoping review was performed by our research group on best practices for engaging in weight-related conversations [10]. Parents also participated in semi-structured interviews to discuss and provide input on topics regarding weight. Themes and messages for the video were subsequently identified and used to develop the video content. These included how to re-direct the focus of weight-related conversations to ones of healthy behaviours, how to respond to your child’s concerns about their weight sensitively, tips to promote healthy lifestyle behaviours, and discussions on weight bias. *VideoScribe (Version 3.2.1 PRO, Sparkol Inc, USA)* [11] and *Graphic Software* [12] were used to develop a high-definition animated whiteboard-style video. Aiming to enhance the learning and overall effectiveness of the video, Mayer’s principles of multimedia design were used to inform video development [13]. Parents of children under the age of 18 were recruited to provide input following viewing of the video. Additional input was provided by health care professionals (HCP) with expertise in pediatrics, eating disorders, and psychology. Usability testing with parents was completed over three cycles to refine the final video.

### Subjects/ data collection

Parents were invited to participate via posters that were displayed throughout SickKids hospital, a ‘tweet’ posted on the SickKids Twitter© page, and by word of mouth (i.e. snowball sampling). All interested participants contacting us by email or phone were provided with a secure internet link via email. This link granted access to the study through the Research Electronic Data Capture (REDCap) portal [14]. Implied consent was provided by completing and submitting the questionnaires, a process which was vetted and approved by the SickKids Institutional Research Ethics Board.

A pre-video questionnaire (Supplementary file 1) was completed by participants to collect demographic information, parental and relationship status, and ratings of perceived self-efficacy. Self-efficacy was measured by asking parents to “rate their degree of confidence on initiating a conversation with their child about weight” and “answering questions or concerns their child may have about their weight”, on a scale ranging from 0 to 100. Parents were then prompted to watch the video. Immediately after watching the video in its entirety, parent perceived level of self-efficacy was re-assessed and satisfaction with the video was evaluated through a questionnaire (Supplementary file 1). Respondents’ perceptions of video length, information presented, and overall satisfaction were included. Parents were also encouraged to comment on the parts of the video that offered the greatest and least usefulness, and suggestions for improvement. Before ending the session, participants were asked whether they would agree to be contacted 4–6 months later to complete a similar assessment of self-efficacy and satisfaction (Supplementary file 2). Pre- and post- questionnaires were adapted from a previous study assessing satisfaction and relevance of a video [15] using questions rated on a 1–5 Likert Scale [16]. Questions on self-efficacy were developed in accordance with the Bandura Guide [17].

### Statistical analysis

Statistical Analysis Software (SAS) version 9.4 (SAS Institute Inc., Carey, NC) was used for this study. Descriptive statistics were used to summarize baseline parent demographics and user satisfaction, and were presented as mean and standard deviations, or median and interquartile range, where appropriate. All continuous variables were tested for normality using a Shapiro-Wilk test. Since perceived self-efficacy scores were non-normal, we used a PROC GLIMMIX procedure to analyze group (“no concern”, “underweight”, “overweight”) and time (pre-, post, and retention [4–6 months later]) and their interaction using a 2-way analysis of variance (ANOVA) on perceived self-efficacy scores. Post-hoc analysis by the Tukey-Kramer test was performed when main and interaction effects were found to be statistically significant. A sample size of 29 achieves 80% power to detect a mean change of 10-points on the scale between pre- and post-video and between pre- and 4 to 6 months later with an estimated SD of 15 and α of 0.13 using a 2-sided paired t-test. To account for potential dropout, a minimum of 35 participants were recruited. A *p*-value less than 0.05 was deemed to be statistically significant.

## Results

### Video development

The five and a half minute educational video addressed weight-related conversations between parents and their children, and was created based on a scoping review and three iterative feedback cycles with parents and HCP experts in psychology, obesity, and eating disorders (i.e anorexia nervosa, bulimia nervosa, etc.) [10]. Three common scenarios between a parent and their child are presented, with emphasis on how to respond to their weight-based concern. The video includes information on timing of these conversations, how to conduct these conversations, and what language to use when addressing weight-based concerns. A focus on healthy lifestyle behaviours, rather than weight, was emphasized, and the need to highlight positive behaviours in their children was underscored. The video concluded with a list of professional health care resources for parents and their children. The video can be viewed at https://meant2prevent.ca/2019/05/01/communicating-about-weight/ [18].

### Description of the participants

**Fig. 1:**
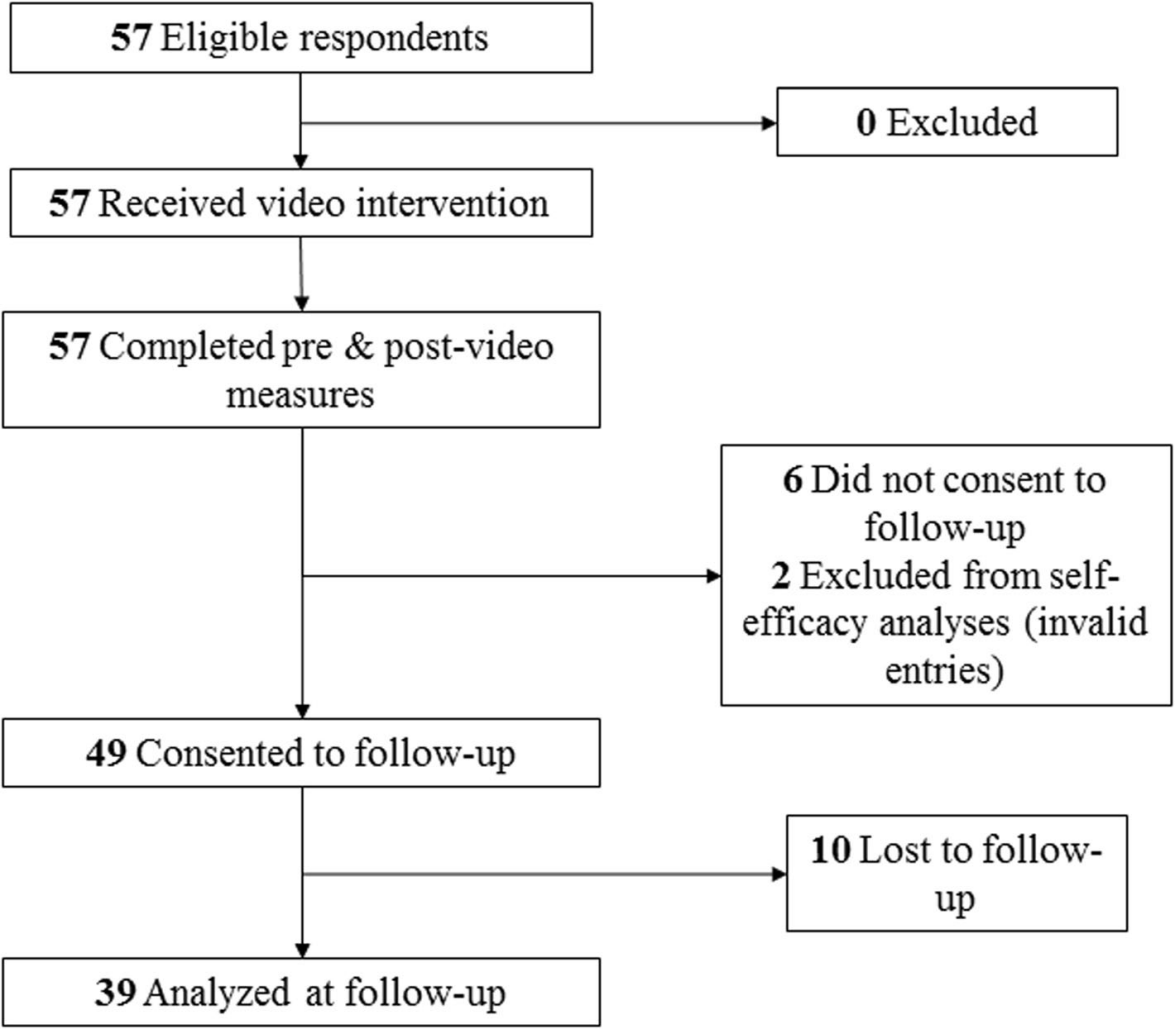
Consolidated Standards for Reporting of Trials (CONSORT) Flow Diagram

A total of 57 respondents (55 female, 96%) completed the baseline assessment, and 39 participated at the 4–6 month follow-up (Fig. 1). Demographic characteristics of the respondents are summarized in Table 1.

**Table 1:** Demographic characteristics of the study participants (*n* = 57)

Characteristics	Number of Participants (%)
**Age (years)**	
18–44	15 (27)
≥ 45	42 (73)
**Sex**	
Female	55 (96)
Male	2 (4)
**Ethnicity**	
White	43 (75)
Other	13 (23)
I prefer not to answer	1 (2)
**Highest Level of Education**	
Did not complete high school	2 (4)
High school graduate or equivalent	2 (4)
College degree	7 (12)
Bachelor degree	20 (35)
Postgraduate degree	26 (46)
**Number of Children**	
One	7 (12)
Two	27 (47)
Three or more	22 (39)
I prefer not to answer	1 (2)
**Marital Status**	
Married or common-law	47 (82)
Widowed	1 (2)
Divorced/separated	9 (16)
**Concerns about own weight**	
Yes (overweight)	29 (51)
Yes (underweight)	0
No	26 (46)
I prefer not to answer	2 (4)
**Concerns about child’s weight (*n*** **= 56**^**a**^**)**	
Yes (overweight)	10 (18)
Yes (underweight)	8 (14)
No	37 (66)
I prefer not to answer	1 (2)

^a^One respondent was excluded due to selection of both Yes (overweight) and Yes (underweight)

### Perceived self-efficacy

At baseline, there were 37 parents with no concerns about their child’s weight, 10 parents with concerns about their child being overweight, and 8 parents with concerns about their child being underweight. At the 4–6 month follow-up, there were 27 parents with no concerns about their child’s weight, 7 parents with concerns about their child being overweight, and 5 parents with concerns about their child being underweight. One participant (Table 1) selected both “Yes (overweight)” and “Yes (underweight)” and was therefore excluded from the self-efficacy analysis.

**Fig. 2:**
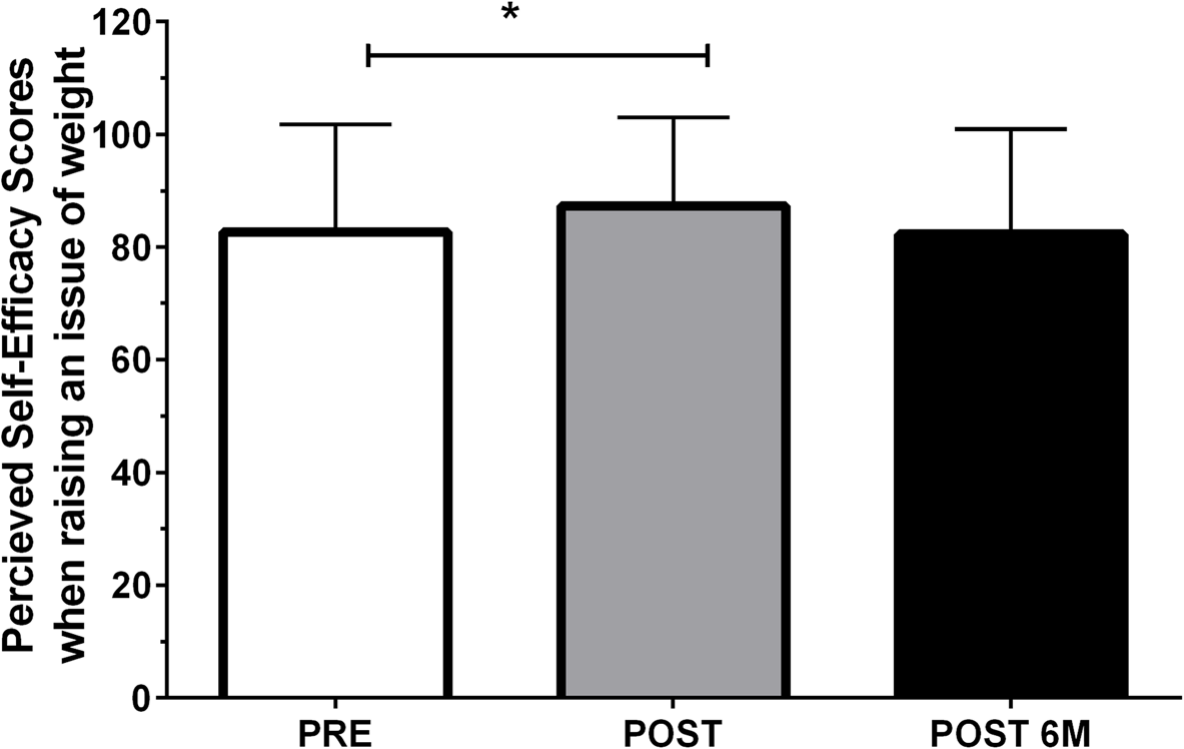
Perceived self-efficacy scores when raising an issue of weight with children at pre- (*n* = 55) and post- video (*n* = 55), and 4–6 months post baseline (*n* = 39). *Significant difference between pre- and post-video perceived self-efficacy score (Tukey-Kramer Post-hoc test *p* = 0.005). Values are reported as mean ± SD. Abbreviations: PRE, pre-video; POST, immediately post-video; POST 6 M, retention 4 to 6 months after watching the video

For self-efficacy in “raising the issue of weight”, there was a main effect of time (*p* = 0.002, Fig. 2), but not group (*p* = 0.220) or a group-by-time interaction (*p* = 0.613). Self-efficacy scores increased pre-to-post video (82.7 ± 19.0 to 87.3 ± 15.7; *p* = 0.005), but scores were not significantly different at the 4–6 month follow-up (82.3 ± 18.6) compared to pre-video (*p* = 0.993) and post-video (*p* = 0.081).

**Fig. 3:**
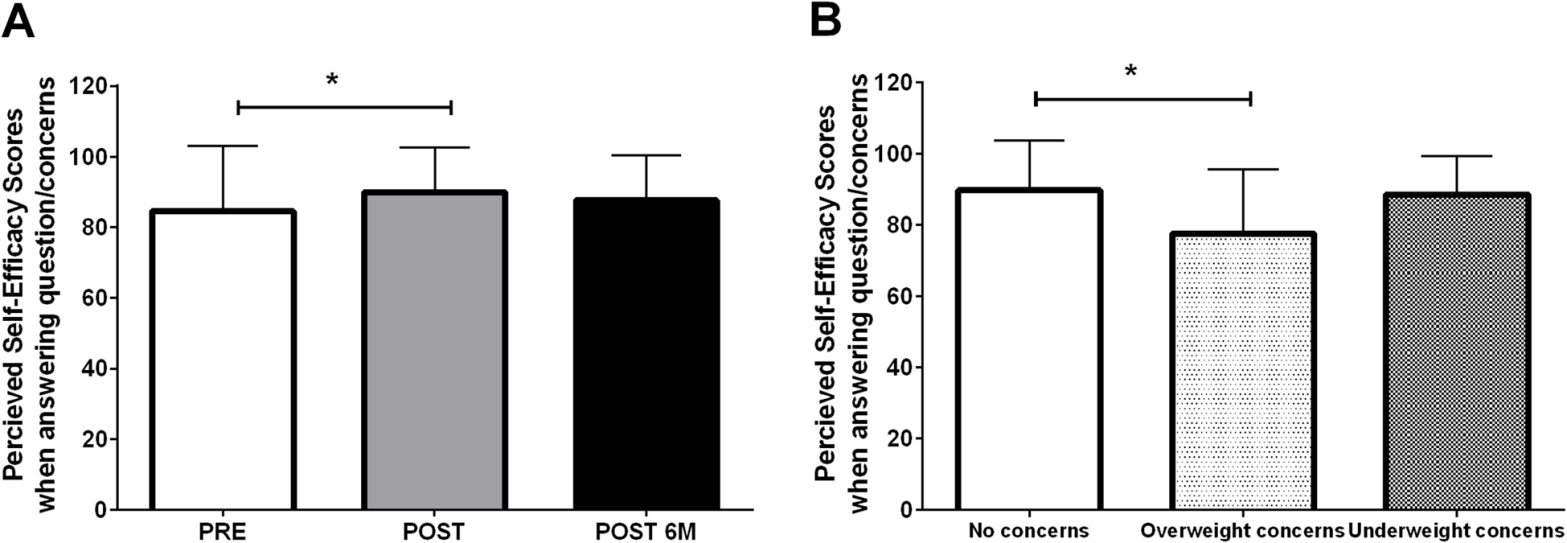
**a** Perceived self-efficacy scores when answering questions or concerns your children may have about their weight at pre- (*n* = 55) and post- video (*n* = 55), and 4–6 months post baseline (*n* = 39). *Significant difference between pre- and post-video perceived self-efficacy score (Tukey-Kramer Post-hoc test *p* = 0.001). **b** Perceived self-efficacy scores when answering questions or concerns your children may have about their weight between parents reporting no weight concerns (*n* = 64), concerns about child being overweight (*n* = 17), and concerns about child being underweight (*n* = 13). *Significant difference between parents reporting no weight concerns and parents reporting concerns about their child being overweight (Tukey-Kramer Post-hoc test *p* = 0.031). Values are reported as mean ± SD. Abbreviations: PRE, pre-video; POST, immediately post-video; POST 6 M, retention 4 to 6 months after watching the video

For self-efficacy in “answering questions or concerns”, there was a main effect of time (*p* = 0.001, Fig. 3a) and group (*p* = 0.039, Fig. 3b), and a trend for a group-by-time interaction (*p* = 0.064). Self-efficacy scores increased pre-to-post video (84.6 ± 18.4 to 90.0 ± 12.6; *p* = 0.001), but scores were not significantly different at the 4–6 month follow-up (87.6 ± 12.8) compared to pre-video (*p* = 0.096) and post-video (*p* = 0.851). Parents with concerns about their child being overweight had significantly lower perceived self-efficacy scores compared to parents with no concerns about their child’s weight (77.5 ± 18.2 vs. 89.8 ± 14.0, *p* = 0.031). Parents with concerns about their child being underweight (88.6 ± 10.7) did not differ in perceived self-efficacy from parents with no concerns (*p* = 0.975) and parents with concerns about their child being overweight (*p* = 0.187).

### Evaluation of the video

**Fig. 4:**
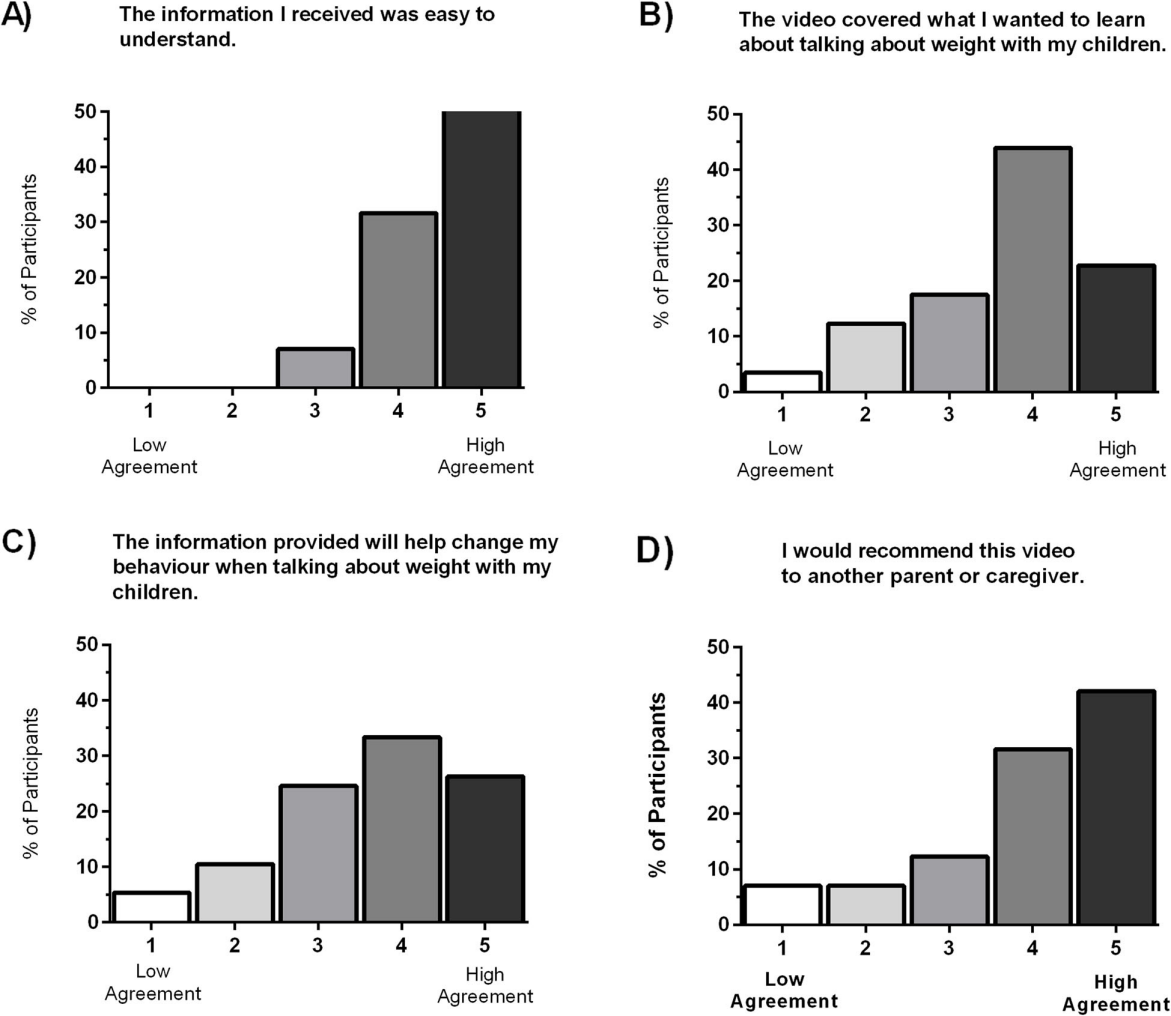
Video Evaluation. Participant agreement (%) measured using a Likert scale (1 to 5, where low agreement = 1 and high agreement = 5) is shown for the following statements (*n* = 57): **a** The information I received was easy to understand, **b** The video covered what I wanted to learn about talking about weight with my children, **c** The information provided will help change my behaviour when talking about weight with my children, and **d** I would recommend this video to another parent or caregiver (*n* = 57)

Overall, the video was well received by parents (Fig. 4). A total of 77% (44 participants) agreed with the statement “the content was presented in an interesting way that held my attention”. Overall, 89% (51 participants) agreed with the statement “the length of the video was appropriate”, and 67% (38 participants) agreed with the statement “I received the right amount of information”.

A very large proportion of respondents, 93% (53 participants) agreed that “the information I received was easy to understand”. Additionally, 77% (44 participants) indicated positive agreement when asked about their satisfaction with the video and 72% (41 participants) found the video enjoyable.

Moreover, 67% (38 participants) indicated that the video covered what they wanted to learn about talking about weight with their children, and approximately half the participants (49%, or 28 participants) learned something new about talking about weight with their children. It was found that 58% (33 participants) agreed the questions they had regarding talking about weight with their children were answered. A total of 60% (34 participants) indicated that the information the video provided would change their behaviour when talking about weight with their children. Lastly, 74% (42 participants) would recommend this video to another parent or caregiver.

Parents also were prompted to indicate which part of the educational video was found to be the most helpful, with the ability to select multiple options (Table 2).

**Table 2:** Participant selection of video components found to be most helpful (*n* = 57)

Video Components	Number of Participants (%)
How to re-direct the focus of weight-related conversations to one of healthy behaviours.	44 (77)
How to respond to your child’s concerns about their weight in a sensitive manner.	37 (65)
Key tips to promote healthy lifestyle behaviours.	25 (44)
Discussions on weight bias (judgements based on a person’s weight).	13 (23)

Among the 40 parents who completed the questionnaire 4–6 months after watching the video, 38% (15 participants) of parents agreed that “the information provided has helped change the way I talk to my children about weight”. When asked again if they would still recommend this video to another parent or caregiver, 58% (23 participants) indicated agreement.

## Discussion

In this study, we developed an educational video based on evidence, parental, and HCP feedback, and assessed its impact on parent’s self-efficacy in communicating about weight-related issues with their children. Parents’ perceived self-efficacy scores for “raising the issue of weight” with their children and “answering questions or concerns” increased significantly immediately after watching the video, however this returned to baseline at follow-up 4–6 months later. Although this effect was not maintained at follow-up 4–6 months later, no significant decline was observed, suggesting skill retention. Interestingly, compared with parents who showed no concern with their child’s weight, parents who had concerns about their child being overweight had significantly lower perceived self-efficacy scores for answering questions or concerns their child may have about their weight. Evaluation of the video indicated that parents were satisfied with its length and content, and a majority of parents felt that the information would change their behaviour.

The need for evidence-based educational resources on how to communicate about weight with their child was identified by parents in focus groups and through a scoping review [4, 7]. Research has also shown that when parents encourage their child to adopt healthy lifestyle behaviours, children are less likely to engage in unhealthy weight loss practices and are less likely to experience depressive symptoms [19]. In a study of 165 mother-daughter dyads where the mothers were actively encouraging their daughters to diet, the child was eight times more likely to diet, but many had unexpected body mass index percentile increases [20]. Emerging evidence also suggests that avoidance of weight-based teasing and negative commentary directed towards the child leads to more favourable psychological outcomes and fewer weight-control behaviours [21–23]. These factors led to the video’s focus on developing healthy habits, rather than a focus on weight. Of note, perceived self-efficacy scores were lower for parents with overweight concerns for their child. Existing literature suggests that parents who view their children as being overweight are associated with their children viewing their body size negatively, leading to unexpected weight gain as a result of failed weight loss attempts [24]. The self-perception of being overweight has also been reported to increase the odds of medium and high psychological distress [25]. Moreover, a systematic review revealed that individual overweight perception was linked to disordered eating and increased weight gain over time, in some study groups [26]. Whether this diminished self-efficacy in parents with overweight concerns for their child translates to changes in behaviour remains to be determined.

To our knowledge, this is the first study to use a video to assess parents’ self-efficacy in initiating weight-related conversation with their children. In a previous study that made usage of a video-intervention program for parents with Prelingual Deaf and Hard-of-Hearing children, video intervention led to increases in parental sensitivity, child responsiveness, and reported self-esteem compared to the non-treatment baseline. It also encouraged more connected parent-child interactions [27]. The majority of our respondents were satisfied with our video’s duration and content (comprehensibility and presentation). These findings are in line with a previous report which found that the most effective educational videos are those that are brief and specific, provide complementary audio and visual elements, have embedded active learning, and have an enthusiastic conversational style [28]. The use of a video for education has additional potential benefits including translatability and inclusivity (narration can be done in different languages and closed captions can be included for the hearing impaired), accessibility, ability to self-pace and view on multiple occasions, as well as cost-effectiveness when compared to in-person teaching [29].

This study is not without limitations. Inclusion of a control cohort could have strengthened our findings. Due to the self-selected nature of the parent recruitment, extrapolation of results to other parent populations may be limited. It is possible that the self-selected recruitment biased participants to those who already had higher self-efficacy due to an interest in the topic, and to those less concerned about their child’s weight. Although we observed an improvement in self-efficacy post-video, we may have underestimated the potential effect of the video to increase self-efficacy. Respondents were relatively homogenous with respect to multiple demographic characteristics. This lack of diversity in the sample may reduce generalizability of the results. This study design makes use of participant self-assessment using a questionnaire, and some individuals may overrate their ability to perform a skill or task, and may have difficulty assessing how well or poorly they comprehend new material [30]. The video was designed to provide a general approach to communicating about weight with children of varying ages; more directed approaches toward younger versus older children and adolescents could be the subject of future work.

The strengths of this study include a 4–6 month follow-up to evaluate the longitudinal effects of the video on perceived self-efficacy. Additionally, the video seemed most effective for parents who identified concerns with their child’s weight, which is important, as these families may be most at risk of negative unanticipated consequences after discussing weight.

## Conclusions

In summary, this pilot study demonstrated improvements in parents’ perceived self-efficacy for initiating weight-related conversations with their children and answering their questions after watching an evidence-based educational video. Future research in other parent populations (e.g. parents of children attending weight management programs, parents of diverse ethnicity etc.) as well as the clinical and behavioural outcomes of children whose parents have watched and successfully implemented the video’s teachings would provide further insight into the potential utility of this educational tool.

## Supplementary information

**Additional file 1.** Parent Questionnaire.

**Additional file 2.** Parent Questionnaire (4–6 Months Post).

## Data Availability

Data are available from the authors upon request.

## Abbreviation

HCP: Health care professional
