# Supplementary material for: Careful conversations: an educational video to support parents in communicating about weight with their children
Source: BMC Pediatr. 2020 Aug 24;20:397. doi: 10.1186/s12887-020-02284-6 (PMC7446051; doi:10.1186/s12887-020-02284-6)
Supplement: Supplementary file 1 — Additional file 1. Parent Questionnaire. [file 12887_2020_2284_MOESM1_ESM.pdf]

Parent Questionnaire

Please complete the survey below.

Thank you!

We are very interested in receiving your feedback about the video, 'Communicating about weight with families'. By completing and submitting this questionnaire you are consenting to be part of this study. Data submitted in the following questionnaire will be collected for the purpose of the study only. The video is 5 minutes long and the questionnaire will take 5 minutes to complete. By completing this questionnaire, you are entered in a draw to win a $100 gift card. Please do not hesitate to contact us (rebecca.noseworthy@sickkids.ca) if you have any questions or concerns.

Thank you!

What is your age?

- ◯ < 18 years old
- ◯ 18-24 years old
- ◯ 25-34 years old
- ◯ 35-44 years old
- ◯ 45-54 years old
- ◯ >55 years old
- ◯ I prefer not to answer

What is your gender?

- ◯ Female
- ◯ Male
- ◯ Other
- ◯ I prefer not to answer

What is your marital status?

- ◯ Single, never married
- ◯ Married or common-law partner
- ◯ Widowed
- ◯ Divorced
- ◯ Separated
- ◯ I prefer not to answer

What is your ethnicity?

- ◯ White
- ◯ Hispanic or Latino
- ◯ Black or African American
- ◯ Aboriginal
- ◯ Asian / Pacific Islander
- ◯ Other
- ◯ I prefer not to answer

Please specify

____

What is the highest degree or level of school that you have completed?

- ◯ Grade 8 or less
- ◯ Some high school, no diploma
- ◯ High school graduate, diploma or the equivalent
- ◯ Trade/technical training
- ◯ College degree
- ◯ Bachelor degree
- ◯ Master's degree
- ◯ Doctorate degree or higher
- ◯ I prefer not to answer

How many children do you have?

◯ 1
◯ 2
◯ 3
◯ 4
◯ >4
◯ I prefer not to answer

Do you have concerns that any of your children may be underweight?

◯ Yes
◯ No
◯ I do not know
◯ I prefer not to answer

Do you have concerns that any of your children may be overweight?

◯ Yes
◯ No
◯ I do not know
◯ I prefer not to answer

Do you have any concerns about your own weight?

◯ Yes (Underweight)
◯ Yes (Overweight)
◯ No
◯ I do not know
◯ I prefer not to answer

Please rate how certain you can do the following tasks right now.

Rate your degree of confidence by recording a number from 0 to 100 using the scale given below:

0	10	20	30	40	50	60	70	80	90	100
Cannot do at all					Moderately can do					Highly certain can do

Raising the issue of weight with your children?

____
(Confidence (0-100))

Answering questions/concerns your children may have about their weight?

____
(Confidence (0-100))

Educational Video:

Please watch the following video, 'Communicating about weight with families - for Parents.'

Please rate how certain you can do the following tasks right now.

Rate your degree of confidence by recording a number from 0 to 100 using the scale given below:

0	10	20	30	40	50	60	70	80	90	100
Cannot do at all					Moderately can do					Highly certain can do

Raising the issue of weight with your children?

____
(Confidence (0-100))

Answering questions/concerns your children may have about their weight?

____
(Confidence (0-100))

There were several components discussed in the video. Please help us understand your opinion on these:

Please check off which part(s) of the video was MOST helpful for you (check all that apply):

☐ Discussions on weight bias (judgments based on a person's weight)
☐ How to respond to your child's concerns about their weight in a sensitive manner
☐ How to re-direct the focus of weight-related conversations to one of healthy behaviours
☐ Key tips to promote healthy lifestyle behaviours

Please check off which part(s) of the video was LEAST helpful for you (check all that apply):

☐ Discussions on weight bias (judgments based on a person's weight)
☐ How to respond to your child's concerns about their weight in a sensitive manner
☐ How to re-direct the focus of weight-related conversations to one of the healthy behaviours
☐ Key tips to promote healthy lifestyle behaviours

With regard to the video you just watched, please indicate your level of agreement with each statement on a scale from 1 (low agreement) to 5 (high agreement).

	1 (Low agreement)	2	3	4	5 (High agreement)
The content was presented in an interesting way that held my attention	○	○	○	○	○
The length of the video was appropriate	○	○	○	○	○
The length of the video is too short	○	○	○	○	○
The length of the video is too long	○	○	○	○	○
I received the right amount of information	○	○	○	○	○
The information I received was easy to understand	○	○	○	○	○
The video covered what I wanted to learn about talking about weight with my children	○	○	○	○	○
I learned something new about talking about weight with my children	○	○	○	○	○
My questions about talking about weight with my children were answered	○	○	○	○	○

The information provided will help change my behavior when talking about weight with my children	◯	◯	◯	◯	◯
I would recommend this video to another parent or caregiver	◯	◯	◯	◯	◯

Overall:
Please indicate your level of agreement with each question below by marking a number between 1 (not at all) and 5 (very).

	1 (Not at all)	2	3	4	5 (Very)
Overall, how SATISFIED were you with the video?	◯	◯	◯	◯	◯
Overall, how ENJOYABLE was the video?	◯	◯	◯	◯	◯

What did you find MOST USEFUL about video? __________________________________

What could have been done DIFFERENTLY to improve your learning about talking about weight with your children? __________________________________

What other IMPROVEMENTS do you have for making this video more helpful and/or interesting? __________________________________

Do you have any other comments or suggestions? __________________________________

Answering yes to any of the below questions will prompt you to provide an e-mail address. This will be used for contact purposes only. All answers provided in the above survey will be exported separately and will not be linked to your contact information.

Please be aware the e-mail is not a secure form of communication as it can be accessed by unauthorized third parties. By providing your e-mail, you confirm that you are aware of this risk.

Can we contact you in 4-6 months to complete a 2-question survey?
◯ Yes
◯ No

By completing this questionnaire, you are entered in a draw to win a $100 gift card. If you win this gift card, can we contact you through email?
◯ Yes
◯ No

Would you like to be contacted by email to receive the summary of this study and view the final video?
◯ Yes
◯ No

Please provide your e-mail address

____

Thank you for participating in our study.

For additional resources you may consult the following links:

1) Centre for Healthy Active Kids:
http://www.sickkids.ca/Centre-for-Healthy-Active-Kids/

2) About Kids Health:
http://www.aboutkidshealth.ca/

Many resources are available at the Hospital for Sick Children:

1) SickKids Team Obesity Management Program (STOMP)
http://www.sickkids.ca/STOMP/

2) Infant and Toddler Growth and Feeding Program
http://www.sickkids.ca/PaediatricMedicine/What-we-do/Paediatric-consultation-clinic/index.html

3) Adolescent Medicine Clinic
http://www.sickkids.ca/AdolescentMedicine/
