# Supplementary material for: Careful conversations: an educational video to support parents in communicating about weight with their children
Source: BMC Pediatr. 2020 Aug 24;20:397. doi: 10.1186/s12887-020-02284-6 (PMC7446051; doi:10.1186/s12887-020-02284-6)
Supplement: Supplementary file 2 — Additional file 2. Parent Questionnaire (4–6 Months Post). [file 12887_2020_2284_MOESM2_ESM.pdf]

# Parent Questionnaire (4-6 Months Post)

Please complete the survey below.

Thank you!

Please do not hesitate to contact us (michele.strom@sickkids.ca) if you have any questions or concerns.

Please rate how certain you can do the following tasks right now.

*Rate your degree of confidence by recording a number from 0 to 100 using the scale given below:*

| 0 | 10 | 20 | 30 | 40 | 50 | 60 | 70 | 80 | 90 | 100 |
|---|---|---|---|---|---|---|---|---|---|---|
| Cannot do at all | | | | | Moderately can do | | | | | Highly certain can do |

1) Raising the issue of weight with your children?

0 50 100

*(Place a mark on the scale above)*

2) Answering questions/concerns your children may have about their weight?

0 50 100

*(Place a mark on the scale above)*

**Thinking back to the video you watched 4 to 6 months ago (Communicating about weight with your children) please indicate your level of agreement with each statement below by marking a number between 1 (low agreement) and 5 (high agreement).**

| | 1 (Low agreement) | 2 | 3 | 4 | 5 (High agreement) |
|---|---|---|---|---|---|
| 3) The information provided has helped change the way I talk to my children about weight | ○ | ○ | ○ | ○ | ○ |
| 4) I would recommend this video to another parent or caregiver | ○ | ○ | ○ | ○ | ○ |

Thank you for participating in our study.

For additional resources you may consult the following links:

1) Centre for Healthy Active Kids:
http://www.sickkids.ca/Centre-for-Healthy-Active-Kids/

2) About Kids Health:
http://www.aboutkidshealth.ca/

Many resources are available at the Hospital for Sick Children:

1) SickKids Team Obesity Management Program (STOMP)
http://www.sickkids.ca/STOMP/

2) Infant and Toddler Growth and Feeding Program
http://www.sickkids.ca/PaediatricMedicine/What-we-do/Paediatric-consultation-clinic/index.html

3) Adolescent Medicine Clinic
http://www.sickkids.ca/AdolescentMedicine/
